# Efforts in surgical site infection surveillance at the Mbouo Protestant Hospital in Cameroon

**DOI:** 10.1186/s12893-025-03229-5

**Published:** 2025-10-03

**Authors:** Christian Doll, Lydie Charlie Ndoho Simo, Honorée Jeulefack, Alaric Tamuedjoun Talom, Lazare Kuate Kamdem, Jean-Blaise Kenmogne, Ghyslaine Bruna Djeunang Dongho, Andrej Trampuz

**Affiliations:** 1 Institute of Tropical Medicine and International Health, Charité - Universitätsmedizin Berlin, Corporate Member of Universität Berlin, Humboldt-Universität Zu Berlin and Berlin Institute of Health, Berlin, Germany; 2 Spine and Scoliosis Center, Hessing Foundation, Augsburg, Germany; 3 Faculty of Sciences and Technology, Evangelical University Institute of Cameroon, Mbouo/Bafoussam, Cameroon; 4 Mbouo Protestant Hospital, Mbouo/Bafoussam, Cameroon; 5 Foumban Njissé Protestant Hospital, Foumban, Cameroon; 6 Evangelical University Institute of Cameroon, Mbouo, Cameroon; 7 Nkafu Policy Institute, Yaoundé, Cameroon; 8 Queensland University of Technology, Brisbane, Australia; 9 Center for Musculoskeletal Surgery (CMSC), Charité - Universitätsmedizin Berlin, Corporate Member of Universität Berlin, Humboldt-Universität Zu Berlin, and Berlin Institute of Health, Berlin, Germany

**Keywords:** Surgical site infection, Africa, Cameroon, Antimicrobial drug resistance, Risk factors, Orthopaedics, General surgery, Obstetrics

## Abstract

**Background:**

Surgical site infections (SSIs) are a significant health concern in low- and middle-income countries. In Africa, up to one-third of patients undergoing surgery may be affected by an SSI, and high rates of antimicrobial resistance (AMR) pose an additional threat. Data on the epidemiology and microbiology of these infections are needed but scarce.

**Methods:**

This prospective, observational, pilot study investigated the incidence, risk factors, and microbial spectrum of SSI. All consenting patients undergoing surgery at Mbouo Protestant Hospital in Cameroon were included. An active SSI surveillance system was established and continued after discharge. Data collection took place from April 2021 to February 2022. Risk factors for SSI and for mortality were recorded as well as microbial data. The SSI incidence and the Odd’s ratios were calculated.

**Results:**

One hundred forty-eight patients were included. The total SSI incidence was 7% (11/148) with 3% (2/67) for Obstetrics/Gynaecology, 3% (1/30) for General Surgery and 16% (8/51) for Orthopaedics/Trauma. About 55% (6/11) of SSI appeared after discharge from hospital. Risk factors for SSI were Orthopaedic/trauma procedure, dirty/infected wounds, high intraoperative blood loss and long duration of the operation. The total postoperative mortality was 3% (4/148) with 0% (0/67) for Obstetrics/Gynaecology, 10% (3/30) for General Surgery and 2% (1/51) for Orthopaedics/Trauma. Mortality risk factors were laparotomy, high ASA class and high age. Multi-resistant Staphylococcus aureus and gram-negative bacteria were the main SSI germs. All (2/2) of the non-AMR SSI wounds healed until the end of the study in contrast to only 25% (1/4) of the AMR SSI, all of the non-healed being orthopaedic AMR SSI.

**Conclusion:**

This pilot study reveals a significant burden of SSIs, AMR and perioperative mortality at a district hospital in Cameroon. Despite its limitations, the study identified critical areas for improvement, including developing adapted guidelines for orthopaedic SSIs, improving the implementation of SSI prevention guidelines, and enhancing perioperative antimicrobial stewardship. These findings emphasise the necessity of further research and targeted interventions in this underrepresented, low-resource setting.

**Trial registration:**

Clinicaltrials.gov NCT05018884, date of registration 17/08/2021, retrospectively registered.

**Supplementary Information:**

The online version contains supplementary material available at 10.1186/s12893-025-03229-5.

## Background

Surgical site infections (SSIs) are an important health concern in low and middle income countries, leading to prolonged hospital stay, increased costs, morbidity, disability and mortality [1, 2]. SSIs can vary in severity (superficial, deep, or affecting organs/spaces) and affect all surgical specialties. Depending on the surgical procedure performed, post-discharge surveillance for SSIs is recommended for between 30 and 90 days [3]. Targeted treatment algorithms for SSIs are particularly important for challenging implant-associated infections. Previous studies have shown that up to one-third of patients undergoing surgery in Africa may be affected by a SSI [1]. High rates of antimicrobial resistance (AMR) in these SSIs [4] complicate the treatment even more. International guidelines for the prevention [5] or diagnosis/treatment [6, 7] of SSIs exist, but most recommendations are drawn from studies in high income countries. The context of low resource settings, like in many (underserved) areas in low and middle income countries, is challenging and may hinder the implementation of these guidelines: Limited financial resources, insufficient market supply, lack of medical infrastructure and health workforce are just some examples [8]. Antibiotics recommended in these guidelines may not be available in resource-limited settings, or may simply be too expensive for a patient to afford. It is important to develop and implement SSI prevention and treatment guidelines adapted to this challenging context in order to reduce this complication. To deploy efficient measures for this low-resource context, data on the epidemiology and microbiology of these infections are needed. This involves adapting prevention measures to the most significant risk factors for surgical site infections in austere settings and providing country-specific recommendations on antimicrobial therapy based on local resistance patterns. However, data on this subject is scarce in low- and middle-income countries [9, 10]. Eleven studies investigating SSI incidence were found in Cameroon [11–22]; only two of these [12, 22] included microbiological data, and none of the studies had an active SSI surveillance system in place for at least 30 days after surgery. Active SSI surveillance is important, however, as more than 30% of SSIs are diagnosed after the patient has been discharged from hospital [11].

This scarcity of SSI data is not only present in Cameroon, but in the whole of Sub-Saharan Africa, especially in regard to microbiological patterns of SSIs [23].

This pilot study aims to investigate the incidence and risk factors of SSIs, as well as the microbial spectrum and resistance patterns, by conducting active surveillance after hospital discharge in a Cameroonian district hospital with a mixed operative spectrum. The results will help prioritise future research according to need and potential for improvement. They will also inform the optimal design of future studies at Mbouo Protestant Hospital in Cameroon and similar settings. This study is one of the few in sub-Saharan Africa to collect microbial SSI data and conduct active surveillance after hospital discharge. It will contribute urgently needed epidemiological data and help reduce SSI in the hospital where it was carried out [24].

In addition to the SSI incidence, microbial data and risk factors, we took the opportunity to additionally investigate the perioperative mortality at the study site as the latter is an important core indicator in the context of Global Surgery [8].

## Methods

### Study design, setting, study population, data collection

This is a prospective, observational, pilot study targeting all consenting patients undergoing surgery at Mbouo Protestant Hospital in Cameroon. Founded in 1928, the hospital forms part of the Evangelical Church of Cameroon's (EEC) health department. It is located just outside Bafoussam, the regional capital of Cameroon's West Region. It is a 200-bed, non-profit, denominational, district-level hospital which is used for referrals in some cases. Around 500 major and 600 minor surgical procedures are performed each year. Major surgical cases are defined as operations performed in the operating theatre under general anaesthesia, spinal/epidural anaesthesia or a similar technique. In contrast, minor surgical procedures such as wound debridement under local anaesthesia, penile circumcision, and complex wound dressing changes in the outpatient surgical procedure room were not included.

All patients that were awaiting a major operation at this hospital or that have been operated in emergency were asked to consent to their participation in the study and were prospectively enrolled thereafter. Patients of all age groups were included in the study, from new-borns to senior citizens, with no age limit. All clean and non-clean (including dirty/infected) major operations were included, independent of the surgical speciality. The pre- and intraoperative data were collected prospectively through active data entry. An active surveillance was performed during 30 days after surgery regarding SSI, mortality and morbidity. In case of a confirmed SSI, the patient was followed up until the healing of the wound (indicative for cure of the infection) and the cases are documented. In the case a patient needed multiple procedures or revision surgery, only the first operation was included; this did affect only patients that needed revision surgery in case of SSI. All included patients received the routine care provided by the health personnel.

Data collection took place between 24th April 2021 and 12th December 2021; follow up started on 24th April 2021 and ended in February 2022.

On a pre-printed data collection form, the following information were recorded:

Age, sex, diagnosis, comorbidities, date of the operation, hospital stay in days before the operation, type of operation (urgent/emergency, semi-urgent within 24 h or elective/planned), hospitalization department (traumatology, gynaecology or surgery and intensive care ward), preoperative hair removal (shaving) performed, preoperative body washing (soap and water) performed, perioperative antibiotics, surgical hand preparation, surgical site preparation, American Society of Anaesthesiologists (ASA) Physical Status Classification, surgical wound classification according to American College of Surgeons (ACS)/Centers for Disease Control and Prevention (CDC) [25], type of anaesthesia (spinal or general), duration of surgery, surgical procedure performed, intraoperative blood loss, air filtration during operation, confirmed SSI, confirmed in-hospital all-cause mortality within 30 days postoperative, confirmed other postoperative complications during hospitalization, date of discharge from the hospital. The pre-operative variables were collected prospectively (for programmed operations) and/or retrospectively (for urgent operations) by extraction from the hospital documentation/logbooks.

The only inclusion criterion was that the patient (including children) underwent surgery at the hospital during the study period, the only exclusion criteria were unconscious or mentally ill patients that were not able to consent.

### Surveillance of SSI and microbiological analysis

An active SSI surveillance system was put in place during hospitalization of the patient and that surveillance continued after discharge from the hospital for a total of 30 days after surgery. During the hospital stay, the wound was regularly checked and evaluated for signs of infections by a trained nurse and/or physician. After discharge from the hospital, a phone call follow up was performed on day 15 and 30 after the operation by a trained study nurse. A standardised questionnaire was used for this follow up. Suspected SSI cases were evaluated and confirmed by a physician and classified according to the type of SSI (superficial, deep, organ/space). The questionnaire in its English version as well as in its French version is available as a supplementary file to this study.

Before the study started, the study coordinator (CD) provided training to the study nurse (LCNS) on the definition and detection of SSIs. The study nurse supervised and conducted SSI surveillance during the patient's hospital stay and carried out post-discharge follow-up by telephone.

For the detection of SSI the definition of SSI events by the CDC [3] was used with the Renz modification for implants [26].

In case of a suspected or confirmed SSI, a bacteriology test was offered to the patient without cost and a swab was taken of the wound at the ward or in the operating room (in case of re-operation). The microbiological culture and antibiotic sensitivity testing were carried out at the laboratory of the Mbouo Protestant Hospital by the local laboratory technicians, using their routinely performed techniques: Bacterial identification was done using biochemical tests (oxidase, catalase, Simmons citrate, Kligler Iron Agar, coagulase and Enterosystem) as well as Gram staining and colony morphology observation. The EUCAST disk diffusion methodology was used with ATCC for quality control. The quality of the performed routine laboratory procedures was assured by a cooperation between the Protestant Hospital Mbouo and the Charité University Medical Centre (Berlin, Germany) through a German Hospital Partnership project [27] as well as by the organisation Biologie Sans Frontières (Lyon, France). After SSI confirmation by the responsible physician, the SSI treatment was continued in accordance with hospital standards, using information from bacteriology and antibiotic sensitivity tests. These patients were followed up until the infection subsided and the wound healed.

### Data processing and statistical analysis

All data from the questionnaires, phone follow up and laboratory testing were collected in an Excel file (Microsoft Corporation). The patient names were coded.

The incidence of SSIs was calculated by the number of patients having developed a SSI divided by the total number of included patients. The postoperative mortality rate has been calculated likewise. The isolated bacteria and their respective antibiotic sensitivity was collected likewise in another Excel file.

The mean/median, percentages and ranges are reported as well as the absolute numbers of the variables, to allow for recognition of missing values.

The statistical analysis was done with R (R Foundation for Statistical Computing). For statistical analysis, the Odds ratios (95% confidence intervals) of the risk factors for SSIs were calculated using the conditional maximum likelihood estimation method. Linear models were fit to estimate the relationship between outcomes and variables of interest. Two-tailed p-values were calculated to determine the statistical significance of the regression coefficients. *P*-values of < 0.05 were considered statistically significant. A statistical check for confounding was performed where applicable. SSI and mortality rates were stratified by procedure and SSI type.

### Ethical considerations

The study was approved by the Cameroon Bioethics Initiative (CAMBIN ERCC), reference number CBI/447/ERCC/CAMBIN, issued 27/11/2019. Written informed consent was sought by every participant before inclusion in the study or by his/hers parents in case of children. Patient data was strictly kept in the study site hospital. Questionnaires and other sheets were pseudonymised to protect the privacy of the patient. Only the authors had access to the full dataset, to evaluate the data as stated in the ethics clearance. The study is registered at clinicaltrials.gov NCT05018884, date of registration 17/08/2021, retrospectively registered.

## Results

### Patient characteristics, surgical procedures and perioperative management

**Fig. 1:**
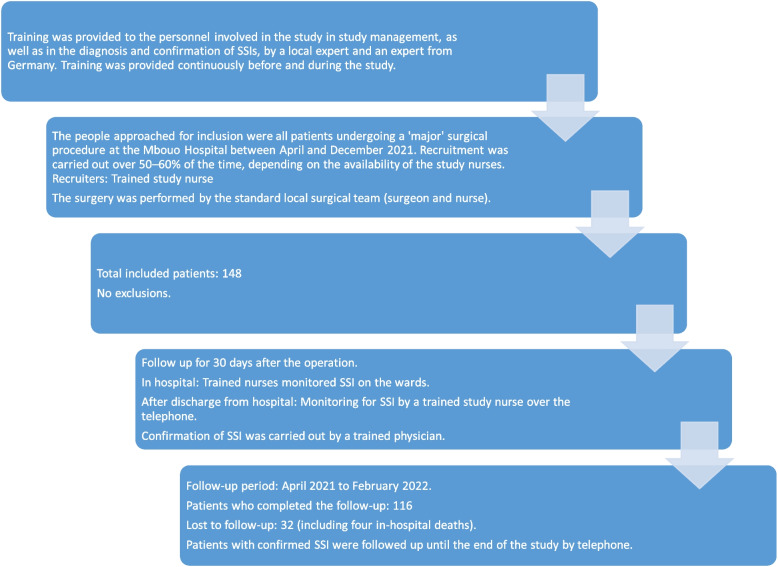
Flowchart of study design, inclusion and follow-up

The inclusion and follow-up of the patients is summarised in Fig. 1.

A total of 148 patients were included with a median age of 31 years and 63% (93/148) females. The ASA Physical Status Classification was 1 for 91% (133/147) of the patients, 2 for 8% (12/147) and 3 for 1% (2/147), respectively. After a median preoperative stay at the hospital of 1 day (range: 0—14 days) the operation was performed. In 59% (87/148) of the cases, the operation was urgent or semi-urgent. The median duration of the operation was 78 min, ranging from 30 to 360 min. Spinal anaesthesia was used in 85% (125/147) of the operations; the rest received general anaesthesia or other types. Surgical hand preparation (first soap/water with optional brush, then alcohol) and disinfection of the surgical site before the operation (first chlorhexidine, then iodized alcohol solution) was carried out as per standard for all patients as well as air ventilation-filtration (filter class EN1822 H13) of the operating room.

Most of the operations were gynaecology/obstetrics procedures (45%, 67/148), followed by orthopaedic surgery with 35% (51/148) and general surgery with 20% (30/148).

Regarding the procedures, in descending order, the most common gynaecology/obstetrics procedures were, caesarean section in 79% (53/67) of the cases, followed by hysterectomy in 8% of cases (5/67), myomectomy and ruptured extra uterine pregnancy.

For orthopaedic/trauma surgery, the most common procedures were osteosynthesis in 71% (36/51) of cases, followed by removal of osteosynthesis material in 12% (6/51) cases, knee prosthesis implantation and debridement/sequestrectomy for osteomyelitis.

The most common general surgery procedures were hernia repair in 43% (13/30) of cases and laparotomy in 37% (11/30); the most common diagnoses for laparotomy were bowel obstruction and gastrointestinal perforation in 55% (6/11) of all laparotomy cases, followed by exploratory laparotomy for peritonitis/abscess, spleen rupture and appendicitis.

Wound class was available for 99% (146/148) of procedures. The wound contamination was class I in 32% (46/146) of the cases, class II in 44% (64/146), class III in 14% (20/146) and class IV in 11% (16/146). About half of the orthopaedic surgery cases presented with wound contamination class III or IV, as well as nearly all laparotomy cases; in contrast to that, nearly all gynaecology/obstetric surgeries and hernia repairs were classified as contamination class II; this difference was highly significant (*p* < 0.001). A detailed evaluation and –if necessary- recoding of the initial wound class values given by the local surgical team was performed during data processing to adhere to CDC criteria [25]; for example, emergency caesarean sections were re-coded as wound contamination class II instead of I.

A perioperative antibiotic was administered in the operating room in 99% (146/148) of the cases. Nearly all gynaecology/obstetrics cases received Ceftriaxone. General surgery cases received either Ceftriaxone, Ampicillin or Ceftriaxone/Gentamicin (+ Metronidazole); of these, hernia cases received either Ampicillin or Ceftriaxone, whereas laparotomy cases received either Ceftriaxone with or without Gentamicin/Metronidazole. 82% (40/49) of the orthopaedic surgery cases received Ceftriaxone only, which was also true for wound contamination class III and IV cases. Antibiotics were generally administered less than hour before incision, as per hospital standard.

After the operation, antibiotics were continued for a median of 4 days in 32% (47/148) of patients. The types of postoperative antibiotics were similar to the aforementioned perioperative ones.

After the operation, the patients stayed hospitalised for a median of 7 days (range: 0—61 days). After discharge from the hospital, it was possible to successfully perform a follow up for 78% (115/148) of the cases. Readmissions occurred only in SSI cases. Post-discharge follow up was performed for all cases by telephone, except for the two 'late' SSI cases that were readmitted at the hospital after several months for revision surgery.

### Surgical site infections

**Fig. 2:**
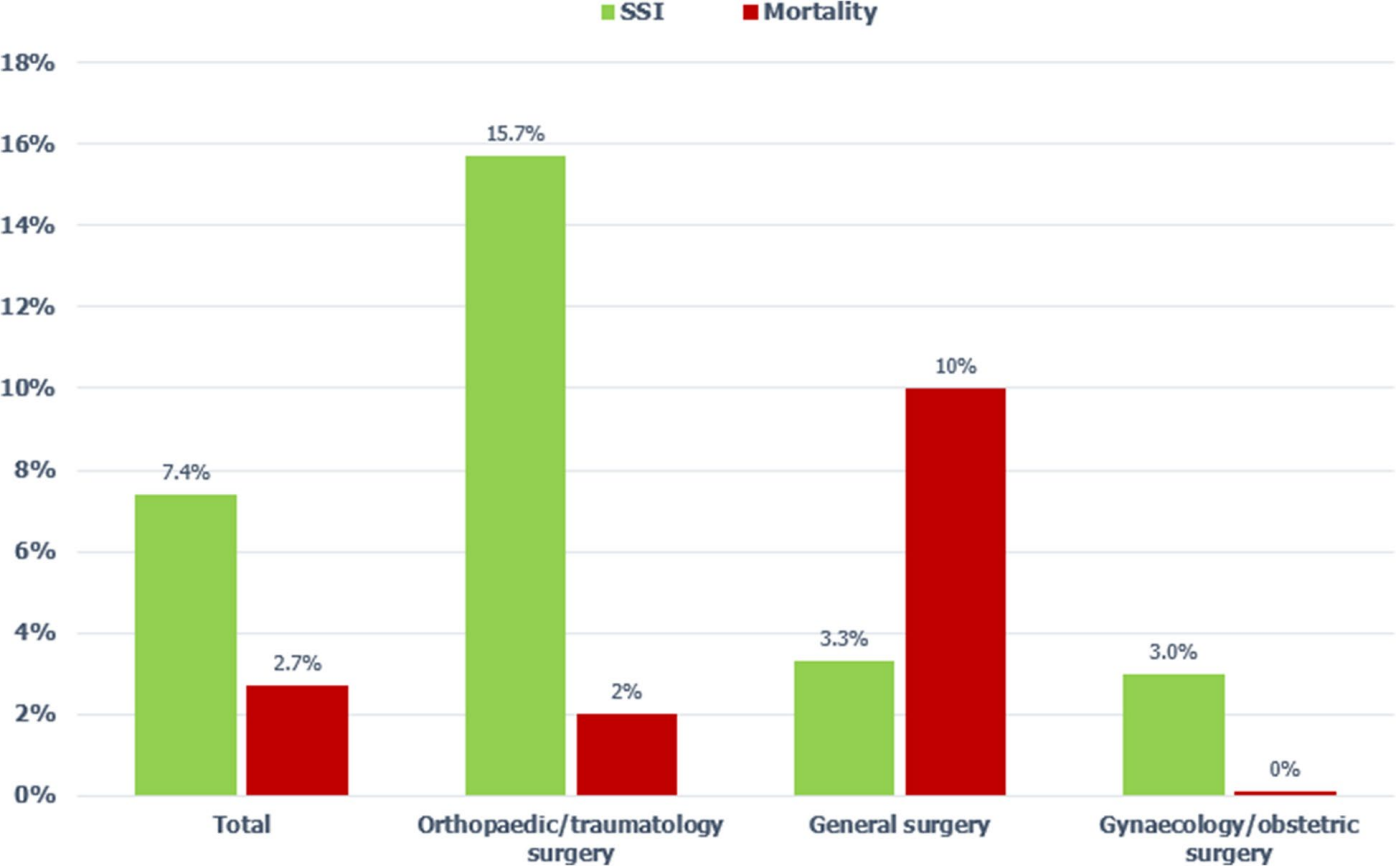
SSI incidence and postoperative all-cause in-hospital mortality rate in total over all patients (left) and by speciality (middle and right)

The total incidence of SSI was 7.4% (11/148). Orthopaedic surgery cases showed the highest incidence with 15.7% (8/51) of all operated patients developing a SSI, followed by general surgery with an SSI incidence of 3.3% (1/30) and gynaecology/obstetrics with 3.0% (2/67), see Fig. 2. Regarding the operative procedures performed, 1.9% (1/53) of all caesarean section cases developed a SSI, 7.1% (1/14) of the other gynaecological cases, none (0/13) of the hernia cases and 9.1% (1/11) of all general surgery laparotomy cases. Statistically significant risk factors for SSI were orthopaedic surgery, high wound contamination class, high intraoperative blood loss and long duration of the operation. The variables are summarized in Table 1.

**Table 1:** Risk factors for SSI of this study

Variable	Total of patients	SSI cases	Proportion of patients who developed SSI (SSI incidence)	Unadjusted/crude OR (95% CI)	*p*-value
Surgical procedure					
Caesarean section	51	1	1.9%	1.0 (ref)	
Hernia repair	13	0	0%	0.0 (0.0–158.6)	n.s
Laparotomy (non-gyn.)	11	1	9.1%	5.0 (0.1–205.6)	n.s
Orthopaedics/trauma	51	8	15.7%	8.5 (1.4–220.1)	0.008
Other gynaecological	14	1	7.1%	3.9 (0.1–158.4)	n.s
Other	6	0	0%		n.s
Surgical speciality					
Gynaecology/obstetrics	67	2	3.0%	1.0 (ref)	
General surgery	30	1	3.3%	1.2 (0.04–15.2)	n.s
Orthopaedics/trauma	51	8	15.7%	5.7 (1.3–42.8)	0.009
Urgency of the operation					
Urgent (immediately)	69	3	4.4%	1.0 (ref)	n.s
Semi-urgent (< 24 h)	18	1	5.6%	1.4 (0.05–12.9)	n.s
Planned	61	7	11.5%	2.8 (0.7–14.0)	n.s
Sex					
Female	93	4	4.3%	1.0 (ref)	
Male	55	7	12.3%	3.2 (0.9–13.1)	n.s
Wound contamination class					
Clean/class I	46	3	6.5%	1.0 (ref)	
Clean-contaminated/class II	64	2	3.1%	0.5 (0.05–3.2)	n.s
Contaminated/class III	20	1	5.0%	0.8 (0.03–7.6)	n.s
Dirty-infected/class IV	16	5	31.3%	6.2 (1.3–36.2)	< 0.001
Intraoperative blood loss					
Less than 500 ml	129	6	4.7%	1.0 (ref)	
500 ml or more	12	4	33.3%	10.0 (2.1–44.4)	< 0.001
Duration of the operation					
Less than 120 min	91	3	3.3%	1.0 (ref)	
120 min or more	45	7	15.6%	5.2 (1.3–26.6)	0.01
ASA Physical Status class					
1	133	9	6.8%	1.0 (ref)	
2	12	1	8.3%	1.3 (0.03–10.7)	n.s
3	2	0	0%	0.0 (0.0–77.8)	n.s
Preoperative hair removal					
Yes	47	3	6.4%	1.0 (ref)	
No	93	7	7.5%	1.2 (0.3–5.9)	n.s
Preoperative body washing					
Yes	39	3	7.7%	1.0 (ref)	
No	105	7	6.7%	0.8 (0.2–4.3)	n.s
Perioperative antibiotics					
Ceftriaxone	120	8	6.7%	1.0 (ref)	
Ampicillin	12	0	0%	0.0 (0.0–6.2)	n.s
Ceftriaxone/Metronidazole	7	1	14.3%	2.3 (0.05–23.3)	n.s
Ceftriax./Metro./Gentamicin	4	1	25.0%	4.6 (0.08–65.1)	n.s
Other type of antibiotic	3	1	33.3%	6.8 (0.1–143.9)	n.s
None	2	0	0%	0.0 (0.0–79.7)	n.s
Type of anaesthesia					
General anaesthesia	20	1	5.0%	1.0 (ref)	
Spinal anaesthesia	125	10	8.0%	1.7 (0.2–75.4)	n.s
Mixed/other	2	0	0%	0.0 (0.0–770.6)	n.s
Age					
Younger than 50 years	121	9	7.4%	1.0 (ref)	
50 years or older	27	2	7.4%	1.1 (0.1–4.5)	n.s

*n*.*s*. not significant, *OR* Odd’s ratio, *CI* confidence interval. The total of patients (148) of each variable was reduced in case of missing values

No statistically significant correlation could be found between SSI incidence and ASA Physical Status Classification, age, sex, urgency of operation, preoperative hair removal, preoperative body washing, type of anaesthesia performed, duration of preoperative hospital stay, and type of perioperative antibiotics.

Orthopaedic surgery wound contamination class I cases showed a SSI incidence of 12% (3/25), class III 8% (1/12) and class IV 33% (4/12). The most common orthopaedic procedures resulting in SSIs were in descending order: Fixation of (infected) non-union, fixation of closed or open fracture, debridement of osteomyelitis and removal of implant. Interestingly, the 5 total hip and total knee replacement surgeries did not result in any SSI in this relatively short follow up period.

The identified SSI occurred between 5 and 126 days after the operation, 55% (6/11) after discharge of the patient from the hospital. SSI in gynaecology/obstetrics and general surgery occurred between 5 and 15 days (median 11 days) after the operation in contrast to orthopaedic surgery SSI where half (4/8) of the SSI appeared between two weeks and one month after the operation and one quarter (2/8) before that and another quarter (2/8) after that (median 23 days). The two 'late' SSI cases had implant-associated infections and came back to the study hospital for revision surgery during the study and follow-up period. These two 'late' SSI cases were therefore detected by in-hospital SSI monitoring.

All SSI in general surgery (1/1) and obstetrics/gynaecology (2/2) were superficial, in contrast to all orthopaedic surgery SSI (8/8) being indicative for deep SSI.

An SSI was positively correlated with a longer postoperative hospital stay (*p* < 0.001) of mean 28 days compared to 10 days without SSI.

### Postoperative mortality

The postoperative all-cause in-hospital mortality was 2.7% (4/148) over all included patients. All deaths were in-hospital, half of the deaths happened on the first day after surgery and the rest of the deaths happened in the first week after the operation. Regarding the surgical speciality, the postoperative mortality was distributed as follows: Obstetrics/gynaecology 0% (0/67), general surgery 10% (3/30) and orthopaedic surgery 2% (1/51), see Fig. 2. This difference was statistically significant (*p* < 0.01).

Patients with ASA Physical Status Classification 2 and 3 showed a significantly higher postoperative mortality of 17% (2/12) respective 50% (1/2) when compared with ASA 1 cases with 0,8% (1/133) (*p* < 0.001). Patients aged 70 years or older showed a significantly higher postoperative mortality than younger age groups (*p* < 0.001). These two variables, however, were positively correlated; meaning that a high patient age is linked to a higher ASA Physical Status Classification and to a higher postoperative mortality.

Regarding the type of anaesthesia performed, general anaesthesia showed a significantly higher postoperative mortality than compared to spinal anaesthesia (*p* < 0.05).

No statistically significant difference was found in postoperative mortality for the variables sex, emergency of the operation, duration of the operation and intraoperative blood loss. Interestingly, SSI did not provoke a higher in-hospital mortality.

### Microbiological analysis

Microbiological cultures could be performed on eight of the eleven identified SSIs, using wound swabs. Two of these cultures did not identify any microorganisms, so a total of six bacterial lineages and their respective antibiotic susceptibility testing results were identified. No culture provided polymicrobial results. Of the six bacteria isolates, four were *Staphylococcus aureus* and two were Gram-negative bacteria: one *Acinetobacter baumanii* and one *Serratia odorifera*. Regarding the antibiotic susceptibility testing results, 3 of the 4 *Staphylococcus aureus* were Methicillin/Oxacillin resistant (MRSA), and one of those was even resistant to Vancomycin. The *Serratia odorifera* was susceptible to Ciprofloxacin. The *Acinetobacter baumanii* showed extensive resistance to all tested antibiotics including Ciprofloxacin; unfortunately, carbapenem resistance was not tested. This means that two-thirds of the bacterial isolates were resistant to important, commonly used antibiotics. The microbial results are summarized in Table 2. Moreover, five of these six bacterial isolates were from SSI cases following orthopaedic surgery; the remaining isolate was MRSA following a caesarean section.

**Table 2:** SSI microbial results and antimicrobial resistance of this study, for *Staphylococcus aureus*, *Serratia orodifera* and *Acinetobacter baumanii*: R is “resistant”, I is “intermediary” and S is “susceptible”; no sensitivitiy testing performed is”- “; the results of each of the 4 Staphylococcus aureus are in separate columns

Resistance against…	Gram-positive bacterial isolates				Gram-negative bacterial isolates	
	**Staphylococcus aureus (n: 4)**				**Acinetobacter baumanii (n: 1)**	**Serratia odorifera (n: 1)**
	**1**	**2**	**3**	**4**		
Penicillin G	R	R	R	-	-	-
Oxacillin	S	R	R	R	R	-
Amoxicilline/Clavulanic Acid	-	-	-	S	R	-
Cefuroxime/Cefoxitin	R	I	R	R	R	-
Ceftriaxone/Cefotaxime	I	S	S	S	R	R
Imipenem	-	R	-	S	-	-
Ciprofloxacin	S	S	R	R	R	S
Vancomycin	-	R	-	I	R	-
Cotrimoxazole	S	R	R	R	R	R
Lincomycin	S	R	R	R	R	-
Erythromycin	-	S	-	R	R	R
Gentamicin	S	R	S	R	R	S
Amikacin	-	I	-	S	R	-
Tetracycline	S	R	S	-	R	-
Nitrofurantoin	-	-	-	-	R	S

### Treatment and outcome of the SSI cases

It was possible to contact 10 of the 11 patients with SSI at the end of the study: All of the 3 gynaecological/obstetric and general surgery infections have completely healed without any additional surgical procedure by treatment with antibiotics. Of the 7 orthopaedic surgery infections, 4 have completely healed with closed wounds and 3 showed still not yet closed wounds but without apparent signs of infection; 3 of these 7 cases needed an additional revision operation. All (2/2) of the non-AMR SSI wounds healed until the end of the study in contrast to only 25% (1/4) of the AMR SSI, all of the non-healed being orthopaedic AMR SSI.

## Discussion

Studies on SSIs are not uncommon in sub-Saharan Africa [1, 4]. However regarding the reporting on AMR in SSIs, a recent meta-analysis [4] found studies for only about one quarter of Sub Saharan African countries, indicating a gap in microbial testing capacity in this region. This is the first study to investigate the incidence and risk factors of SSIs, as well as the microbial spectrum and resistance patterns, using active surveillance after hospital discharge in Cameroon. It includes a mixed spectrum of patients and surgical procedures at a district hospital.

The results of this pilot study inform the prioritisation of future research according to need and potential for improvement. It makes recommendations regarding the optimal design of such studies, particularly at Mbouo Protestant Hospital in Cameroon and in similar settings.

### Limitations

This study has been carried out at a single hospital. However, it can still contribute to urgently needed data.

This study faced several challenges typical for low-resource settings [28, 29]:

- Study design: Due to budget constraints and limited local personnel, the focus had to be on what was doable and affordable while still ensuring sufficient statistical power. As the hospital was in the process of implementing the WHO 'Global Guidelines for the Prevention of Surgical Site Infection' [5], one focus of the study was to measure key WHO-recommended SSI prevention variables, such as hair removal, antibiotics, surgical hand preparation, and surgical site preparation. An in-depth study of the specific characteristics of subgroups (e.g. delayed caesarean sections, delayed skin suturing and implant infections) was beyond the scope of this pilot study.
- Limited post-discharge surveillance: The aforementioned challenges meant that the study had to limit both the follow-up period and the number of participants. This was done after careful consideration of the potential disadvantages: According to CDC criteria, a follow-up period of 90 days would have been necessary for less than one-third of the included study participants. However, this may have resulted in missed implant-associated infections. With a sample size of around 100, it was expected that the most important variables would have acceptable statistical power.
- Patient recruitment and availability of qualified staff: From roughly 300 patients that were operated in the study period at Mbouo Protestant hospital about half could be included in the study. The main reason was that the local personnel at the hospital involved in patient recruitment were sometimes busy with regular patient care, due to shortage of healthcare workers. Patient recruitment was therefore performed in an estimated 50 to 60% of days in the study period. Other minor reasons were, in descending order of importance: refusal to participate, rapid discharge after the operation before inclusion and peri-operative death after emergency operation before inclusion. The latter should not have been a relevant bias, because the mortality in this study (3%) is in the same range as the peri-operative mortality measured at the Mbouo hospital from 2017 to 2019 (2.1%; unpublished data). The sample in this study is representative compared to this hospital’s operation log books of previous years.
- Data quality: Some values of certain variables were missing. Possible reasons are: incomplete surgical logbooks (for missing intra-operative data), inclusion of the patient in the study after an emergency operation (for missing pre-operative data) and early post-operative patient discharge or mortality (for missing post-operative data). Continuous training of the study personnel was performed before and during the study to reduce missing variables and improve data quality. This helped that less than 10% of the values of each in-hospital variable was missing; often even no value was missing.
- Patient retention and loss-to-follow-up: It was possible to perform post-discharge follow up for 78% (116/148) of the included participants. Reasons for this 22% loss to follow up may be mortality (in-hospital and after discharge) and technical difficulties (missing telephone number, suboptimal rural mobile network coverage, etc.).
- SSI diagnostic and laboratory testing: Of the 11 SSI cases, only six bacterial isolates could be identified. This was due to the wound being dry at the time of the swab, no swab being performed, or the cultures being sterile. As all bacterial cultures were monomicrobial, it remains challenging to culture and identify multiple bacterial lineages in that setting, even after improvements to laboratory quality.

### Strengths

One of the strengths of this study is that an active SSI surveillance system was used and a follow up of the patient after discharge of the hospital was performed. As more than half of the SSI of the present study appeared after discharge, this follow up was important.

Another strength is that microbial data were collected for the SSI in the present study. In Sub Saharan Africa only about half of all SSI studies provided microbial data [23].

Other Cameroonian studies investigating SSIs mostly target regional or tertiary hospitals. As a large quantity of operations are carried out at district level hospitals, this study will contribute to SSI and microbial data needed also for this health system level.

### Surgical site infections

Globally, gastrointestinal and some orthopaedic procedures pose higher SSI risks in low-income settings [30, 31], although data for orthopaedic surgery in low and middle income countries remain scarce.

Compared to high-income countries [5], the current study's SSI rate appears elevated, but lower than the ~ 10% found in a pan-African study [32]. A meta-analysis reported 15% overall SSI incidence in Sub-Saharan Africa, with 19.1% in general surgery, 14.8% in orthopaedics, and 8.6% in gynaecology/obstetrics [23]. The Mbouo Protestant Hospital’s rates were lower for general surgery and gynaecology/obstetrics, possibly due to adherence to WHO SSI prevention guidelines [5]. Risk factors such as long surgery time and high contamination class were confirmed.

Based on a recent meta-analysis of microbial SSI data in sub-Saharan Africa [4], four studies with comparable methodology to that of the present study were identified: single-centre; mixed surgical types; prospective design with post-discharge follow-up; ASA status not restricted; inclusion of only SSI and no other nosocomial infections or wounds; and inclusion of AMR data. However, all were conducted at larger hospitals than the district-level Mbouo Protestant Hospital. Three of these studies come from different hospitals in Ethiopia [33–35] and another one from Rwanda [36]. In comparison, the results of this study at Mbouo Protestant Hospital appear to be consistent with those of other studies in a similar context, albeit with a slightly lower SSI incidence.

In Cameroon, SSI incidence varies widely between 6–31% depending on location [11–21]. In summary, Mbouo Protestant Hospital’s rates were comparable to referral hospitals in the capital cities Douala and Yaoundé but lower than in similar peripheral hospitals.

In this study, orthopaedic SSI rates were higher than other surgical specialities. Sub-Saharan data show orthopaedic SSI rates of 10–23%, rising with wound contamination: 4–10% (class I), 23–27% (class II), 42–50% (class III) and 67% (class IV) [37–40]. Mbouo Protestant Hospital had slightly higher class I and lower class III/IV SSI rates.

SSI also led to longer hospital stays, consistent with prior findings [1].

### Perioperative antibiotic administration

The high observed rate (99%) of perioperative antibiotic administration is consistent with global evidence-based recommendations [41] and WHO recommendations for caesarean sections. [42]. Given the high proportion of caesarean sections, orthopaedic surgeries involving implants and hernia repair, at least 94% (137/146) of the surgeries/diagnoses have recommendations on perioperative antibiotic administration.

Ceftriaxone is the most commonly used antibiotic at the study hospital, as it is at many other hospitals in Cameroon. Reasons for using ceftriaxone instead of first-generation cephalosporins in caesarean sections as recommended by the WHO, may include its consistent local availability and affordability, as well as local concerns about AMR.

The high rate of postoperative continuation of antibiotics is consistent with other studies, although it is known that this practice does not reduce SSIs but increases AMR [41].

### Microbial spectrum and susceptibility

We mainly isolated *Staphylococcus aureus*, followed by Enterobacteriaceae (*Serratia odorifera*) and *Acinetobacter baumannii*. These results are consistent with those from other low- and middle-income countries, where common SSI pathogens include Enterobacteriaceae, *Escherichia coli*, *Staphylococcus aureus* and *Pseudomonas* species [9]. A similar pattern has been observed in sub-Saharan Africa, but data is scarce [1]. *Staphylococcus aureus* was also prevalent in SSIs at the Deido District Hospital in Cameroon [22]. However, unlike in this study and at Yaoundé University Teaching Hospital [12], we did not identify *Pseudomonas* or *Escherichia coli*, nor any *Klebsiella* lineages.

We found that *Staphylococcus aureus* was the most prevalent pathogen in orthopaedic SSIs, which is consistent with worldwide data. In contrast to studies from high-income countries [6, 7], *Staphylococcus aureus* was not only the main pathogen in early but also in late orthopaedic SSI, though the limited sample size (*n* = 5) of our study limits the conclusions we can draw. In contrast to what was expected for open fracture cases [43], we did not find any polymicrobial cases, possibly due to laboratory limitations. Our microbial results are consistent with those of other studies on SSIs in orthopaedic surgery in sub-Saharan Africa [37–40, 44], although we did not identify any *Pseudomonas* lineages.

The antimicrobial resistance patterns at Mbouo Protestant Hospital are consistent with the results of other studies in Africa [45], which revealed an increase in MRSA and multidrug resistance, particularly among Gram-negative bacteria. The local resistance patterns present in nearby healthcare facilities and the regional environment [46], with *Staphylococcus* isolates that are highly resistant to oxacillin, cotrimoxazole and vancomycin, reflect the antibiotic susceptibility of SSIs at Mbouo.

### Postoperative mortality

Despite having younger, lower-risk patients, Africa’s surgical mortality rate (2.1%) is double the global average [32]. Mbouo Protestant Hospital's peri-operative mortality rate of 2.7% reflects this trend, with low death rates observed in caesarean sections and orthopaedic surgeries, and higher rates in gastrointestinal surgeries. Acute intestinal obstruction was the main cause of death, in line with regional findings [47]. Although SSI raises mortality in low- and middle-income countries [2, 9], this was not observed here, likely due to low case numbers (*n* = 4). Therefore, the mortality results of the present study should not be over-interpreted statistically.

### Recommendations

This pilot study identified four key areas for improvement and further research at Mbouo Protestant Hospital:

Firstly, SSIs were most prevalent and complicated in orthopaedic surgeries, indicating the need for targeted efforts regarding musculoskeletal implant-associated infections. Although there are evidence-based guidelines for diagnosing and treating SSIs in orthopaedic surgery [6, 7], these are not adapted to challenging, low-resource contexts, where factors such as the cost and availability of antibiotics and costly revision surgeries can be difficult to overcome. Therefore, future efforts should focus on developing guidelines for treating orthopaedic infections at Mbouo Protestant Hospital, like the Cameroonian multi-centre study ADAPT-OS, which is due to begin in September 2025. Studies of orthopaedic SSIs should pay attention to the sampling and processing of microbial specimens [48], and should stratify by wound contamination class, diagnosis, presence of implants, and operative procedure. Post-discharge surveillance should last at least 3 months and ideally up to 12 months to enable the sufficient detection of implant-associated infections.

Secondly, as SSI rates remain high, the implementation of evidence-based recommendations for SSI prevention [5] must be emphasised further. Cost-effective prevention interventions are already known to lower SSI rates in Africa by 30–60% [49, 50]. Urgent essential surgical services should continue despite high SSI rates, provided that prevention interventions are implemented, as scaling them up is necessary [8]. For non-urgent and non-vital procedures involving considerable risk in low-resource settings, including prosthesis surgery, research into the risk profile is recommended to determine whether the benefits of local surgery outweigh the risks against referral. An interesting area that was beyond the scope of this study is the risk of contamination with delayed amniotic membrane rupture, which demands antibiotics and careful observation of the timing of delivery. More research is needed into delayed primary skin closure, especially in contaminated or dirty wounds [51]. Further research is needed on ventilation/air filtration in operating rooms [5] and improvements to the sterilisation department.

Thirdly, although the number of isolates was small, the AMR findings were notable and warrant further investigation. Further studies and interventions are recommended to improve adherence to evidence-based guidelines on peri-operative antibiotics [41, 42], rather than taking a 'one-size-fits-all' approach with ceftriaxone. This could be achieved by implementing antibiotic stewardship programmes, infection prevention and control measures, and enhanced infection surveillance systems, as well as training personnel in the appropriate use of antibiotics [52, 53]. Surgeons should lead the global fight against AMR in surgery [54]. The recently published African treatment guidelines for common bacterial infections are an important initiative by the Africa Centres for Disease Control and Prevention, aiming to standardise treatment, improve the quality of care, and tackle AMR [55].

Fourthly, although the sample size was small, the high rate of postoperative mortality highlights systemic challenges that should be addressed in future initiatives and studies, particularly in high-risk abdominal surgery. The recently published 'Global Clinical Pathways for Patients with Intra-Abdominal Infections' have the potential to improve patient outcomes in low- and middle-income countries [56]. Reducing mortality in abdominal surgery appears to hinge on early recognition of complications, prompt physiological stabilisation of critically ill patients, and adequate source control and appropriate antimicrobial therapy in cases of infection.

## Conclusion

This pilot study sheds light on the incidence, risk factors, microbial spectrum and AMR patterns of SSIs at a district hospital in Cameroon. Despite its single-centre design and modest sample size, the study provides valuable data on a level of healthcare that is often underrepresented in the literature. Four key areas for improvement and further research at Mbouo Protestant Hospital (and similar settings) were identified:

1. The high burden of SSIs following orthopaedic surgery, characterised by late onset, a high degree of AMR and low wound healing rates. Adapted treatment guidelines for orthopaedic surgery infections should be developed. 2. Although SSIs at the study site are lower than the average for sub-Saharan Africa, they pose a significant risk. The implementation of the WHO Global Guidelines for the Prevention of SSI must be emphasised further. 3. The high AMR observed in SSIs, albeit based on a small number of isolates, signals the need for enhanced peri-operative antimicrobial stewardship. 4. The relatively high perioperative mortality rate at Mbouo Protestant Hospital is consistent with the results of other studies conducted on the African continent. This highlights the urgent need for further research and action in this area.

## Supplementary Information

Supplementary Material 1.

Supplementary Material 2.

## Data Availability

The datasets used and analysed during the current study are available from the corresponding author on reasonable request.

## Abbreviations

AMR: Antimicrobial resistance
ASA: American Society of Anesthesiologists Physical Status Classification
CDC: Centers for Disease Control and Prevention
MRSA: Methicillin resistant Staphylococcus aureus
SSI: Surgical site infection
WHO: World Health Organization
