# Supplementary material for: Efforts in surgical site infection surveillance at the Mbouo Protestant Hospital in Cameroon
Source: BMC Surg. 2025 Oct 3;25:419. doi: 10.1186/s12893-025-03229-5 (PMC12492840; doi:10.1186/s12893-025-03229-5)
Supplement: Supplementary file 1 — Supplementary Material 1. [file 12893_2025_3229_MOESM1_ESM.pdf]

Surveillance of Surgical Site Infections (SSI)

NB: sheet to fill out during each change of wound dressing; sheet to be given to the operating room directly after discharge of the patient from the hospital; doctor to inform if infection

Name : _____________________ **First name : ____________________**

Hospital code: ______________

Telephone number 1 : ____________________ Telephone number 2 : _____________________

Date of operation : ___/___/_____

Date of discharge of patient from the hospital : ___/___/_____ Patient died ☐

Complications (pneumonia, thrombosis, infections….) :☐ _____________________

Evaluation of the wound (during every change of wound dressing !) :

Days after operation	Wound is dry and closed ? (Yes / No)	Symptomes of infection (drainage, redness, swollen, …)? (Yes / No)	Person-in-charge of wound dressing change with signature

Evaluation of a wound infection:

A0. Drainage bloody-serous / serous / transparent / obviously non-infected ☐

A1. Drainage infected / wound draining pus ☐ Date :

A2. Drainage infected / drain draining pus ☐ Date :

A3. Absces of surgical site or of operated organe ☐ Date :

A4. Osteosynthesis implant visible (plate etc.) or touchable ☐ Date :

B. Suture loosened / wound incised / deliberated opening of the wound ☐ Date :

C1. Pain (with pressure) of surgical site ☐ Date :

C2. Swelling of surgical site ☐ Date :

C3. Erythema (redness) of surgical site ☐ Date :

C4. Hyperthermia (heat) of surgical site ☐ Date :

C5. Fever (> 38°C) ☐ Date:

D. Doctor confirmed a surgical site infection ☐ Date :

Deepness of the infection:

Superficial (skin and sub-cutaneous tissue) ☐

Deep (fascia, muscle, bone) ☐

Very deep : Organe / space (uterus, abdominal cavity, pleura…) ☐
