# Supplementary material for: Efforts in surgical site infection surveillance at the Mbouo Protestant Hospital in Cameroon
Source: BMC Surg. 2025 Oct 3;25:419. doi: 10.1186/s12893-025-03229-5 (PMC12492840; doi:10.1186/s12893-025-03229-5)
Supplement: Supplementary file 2 — Supplementary Material 2. [file 12893_2025_3229_MOESM2_ESM.pdf]

Surveillance des Infections du Site Opératoire (ISO)

NB : fiche à remplir pendant chaque changement de pansement ; fiche à remettre au bloc opératoire directement après décharge du patient de l'hôpital ; médecin à informer si infection

Nom : _____________________ **Prénom :** ____________________
Code hospitalier: ______________
Numéro téléphone 1 : ____________________ Numéro téléphone 2 : _____________________
Date de l'opération : ___/___/_____
Date de décharge du patient de l'hôpital : ___/___/_____ Patient décédé ☐
Complications (pneumonie, thrombose, infections….) : ☐ _____________________

<u>Evaluation de la plaie (pendant chaque changement de pansement !) :</u>

Jour post-opératoire	Plaie est sèche et fermée ? (Oui / Non)	Symptômes d'une infection (écoulement, aspect rougeâtre, gonflé, …)? (Oui / Non)	Responsable du pansement avec signature

<u>Evaluation d'une infection de la plaie:</u>
A0. Ecoulement séro-sanguineux / séreux / transparent / apparemment non-infecté ☐
A1. Ecoulement infecté / pus sortant de la plaie ☐ Date :
A2. Ecoulement infecté / pus sortant du drain ☐ Date :
A3. Abcès du site opératoire ou d'organe opéré ☐ Date :
A4. Implant d'ostéosynthèse visible (plaque etc.) ou touchable ☐ Date :

B. Sutures lâchés / plaie incisé / ouverture délibérée de la plaie ☐ Date :

C1. Douleur (à la pression) du site opératoire ☐ Date :
C2. Gonflement du site opératoire ☐ Date :
C3. Erythème (rougissement) du site opératoire ☐ Date :
C4. Hyperthermie (chaleur) du site opératoire ☐ Date :
C5. Fièvre (> 38°C) ☐ Date:

D. Médecin a confirmé une infection du site opératoire ☐ Date :

<u>Profondeur de l'infection :</u>
Superficiel (peau et tissue sous-cutanée) ☐
Profond (fascia, muscle, os) ☐
Plus profond : Organe / cavité (utérus, cavité abdominale, pleuvra…) ☐
